# AI-powered skin spectral imaging enables instant sepsis diagnosis and outcome prediction in critically ill patients

**DOI:** 10.1126/sciadv.adw1968

**Published:** 2025-07-18

**Authors:** Silvia Seidlitz, Katharina Hölzl, Ayca von Garrel, Jan Sellner, Stephan Katzenschlager, Tobias Hölle, Dania Fischer, Maik von der Forst, Felix C. F. Schmitt, Alexander Studier-Fischer, Markus A. Weigand, Lena Maier-Hein, Maximilian Dietrich

**Affiliations:** ^1^ Division of Intelligent Medical Systems (IMSY), German Cancer Research Center (DKFZ), Heidelberg, Germany.; ^2^ Helmholtz Information and Data Science School for Health, Heidelberg/Karlsruhe, Germany.; ^3^ Faculty of Mathematics and Computer Science, Heidelberg University, Heidelberg, Germany.; ^4^ National Center for Tumor Diseases (NCT), NCT Heidelberg, a partnership between DKFZ and university medical center Heidelberg, Heidelberg, Germany.; ^5^ Medical Faculty, Department of Anesthesiology, Heidelberg University Hospital, Heidelberg University, Heidelberg, Germany.; ^6^ Department of General, Visceral, and Transplantation Surgery, Heidelberg University Hospital, Heidelberg, Germany.; ^7^ Department of Urology and Urosurgery, Medical Faculty of the University of Heidelberg, University Medical Center Mannheim, Mannheim, Germany.; ^8^ Division of Intelligent Systems and Robotics in Urology (ISRU), German Cancer Research Center (DKFZ), Heidelberg, Germany.; ^9^ DKFZ Hector Cancer Institute at the University Medical Center Mannheim, Mannheim, Germany.; ^10^ Heidelberg University Hospital, Surgical Clinic, Surgical AI Research Group, Heidelberg, Germany.

## Abstract

With sepsis remaining a leading cause of mortality, early identification of patients with sepsis and those at high risk of death is a challenge of high socioeconomic importance. Given the potential of hyperspectral imaging (HSI) to monitor microcirculatory alterations, we propose a deep learning approach to automated sepsis diagnosis and mortality prediction using a single HSI cube acquired within seconds. In a prospective observational study, we collected HSI data from the palms and fingers of more than 480 intensive care unit patients. Neural networks applied to HSI measurements predicted sepsis and mortality with areas under the receiver operating characteristic curve (AUROCs) of 0.80 and 0.72, respectively. Performance improved substantially with additional clinical data, reaching AUROCs of 0.94 for sepsis and 0.83 for mortality. We conclude that deep learning–based HSI analysis enables rapid and noninvasive prediction of sepsis and mortality, with a potential clinical value for enhancing diagnosis and treatment.

## Teaser

AI-driven hyperspectral imaging can rapidly and noninvasively diagnose sepsis and predict mortality in critically ill patients.

## INTRODUCTION

Sepsis is defined as a life-threatening organ dysfunction resulting from a dysregulated host response to infection (*1*). It represents a leading cause of mortality and critical illness worldwide, accounting for 19.7% of global deaths in 2017 (*2*). As the clinical diagnosis of sepsis relies on the presence of organ dysfunction, only patients in advanced stages of the sepsis syndrome are typically identified (*1*). The resulting delay in sepsis diagnosis is critical as the risk of mortality escalates with each hour of treatment delay because of irreversible organ damage (*3*). Conversely, patients incorrectly presumed to have sepsis are often treated unnecessarily with antibiotics, which carry risks ranging from mild side effects to severe complications while simultaneously contributing to the development of multidrug-resistant organisms (*4*, *5*). A critical aspect of sepsis management is the early and accurate diagnosis before the onset of persistent organ dysfunction. This task is complicated by the nonspecific signs and symptoms of the sepsis syndrome, along with the complex, heterogeneous, and not yet fully understood sepsis pathophysiology (*6*). A particular challenge lies in distinguishing between critically ill patients with and without sepsis in the intensive care unit (ICU) because of the higher baseline illness severity and frequent organ failure from both septic and nonseptic inflammation (*7*).

Beyond the early identification of patients with sepsis, the early and accurate identification of ICU patients at high risk of death is crucial. This is because it can substantially improve individual patient outcomes by enabling the timely implementation of appropriate interventions, thereby enhancing patient care (*8*). Moreover, it has the potential to improve the overall efficiency and effectiveness of critical care delivery. This could be achieved through an optimized allocation of limited resources, informed decisions regarding palliative care, and a deeper understanding of the factors that influence patient outcomes (*8*, *9*).

Over the past decades, considerable research efforts have focused on identifying biomarkers for sepsis diagnosis and mortality prediction, with more than 250 molecules proposed as potential diagnostic or prognostic markers. However, to date, no single biomarker has demonstrated outstanding sensitivity and specificity for detecting sepsis and predicting mortality (*10*).

More recently, studies have investigated the use of machine learning to predict sepsis and mortality on the basis of high-dimensional clinical data extracted from electronic health records (EHRs) (*11*). Despite promising performance metrics reported in research studies (*12*, *13*), the clinical translation of EHR-based sepsis and mortality prediction models faces substantial challenges. EHR data, which are primarily collected for the purpose of clinical documentation and billing, lack standardization and contain inaccuracies and biases (*14*). These factors can lead to limited generalizability on external datasets, as demonstrated for several EHR-based sepsis prediction models (*15*, *16*). Furthermore, while EHR adoption is widespread in high-income countries, it lags in low- and middle-income countries (LMICs), where 85% of sepsis cases occur (*2*, *17*).

In recent years, imaging methods such as sublingual microscopy, laser Doppler flowmetry, laser speckle contrast imaging, near-infrared spectroscopy, and hyperspectral imaging (HSI) have revealed that microcirculatory dysfunction, characterized by local zones of hypoxia (*18*, *19*), develops early during sepsis (*20*) and is a key driver of organ failure and poor outcomes (*21*, *22*). We therefore hypothesize that HSI could enable automated sepsis diagnosis and mortality prediction in ICU patients by monitoring microcirculatory dysfunction and edema formation. The key strengths of HSI include its mobile, noninvasive, rapid, objective, cost-effective, and standardized assessments. Unlike other imaging modalities capable of monitoring microcirculation, medical device–graded HSI systems have begun to emerge, paving the way for HSI to become a routine clinical tool (*23*, *24*). While recent initial work on HSI-based sepsis diagnosis performed by ourselves (*25*) and others in parallel (*26*) showed promising performance, all studies conducted so far come with the major limitation that patients with sepsis were compared to healthy volunteers or selectively chosen cohorts, such as patients undergoing pancreatic surgeries (*23*, *27*, *28*). Hence, the proposed algorithms are at high risk of shortcut learning because of confounders such as substantial age gaps and differences in comorbidities and therapies between patients with sepsis and nonseptic controls (*25*). Consequently, they are unlikely to generalize well to realistic clinical applications, such as automated sepsis diagnosis in critically ill ICU patients.

**Fig. 1.**
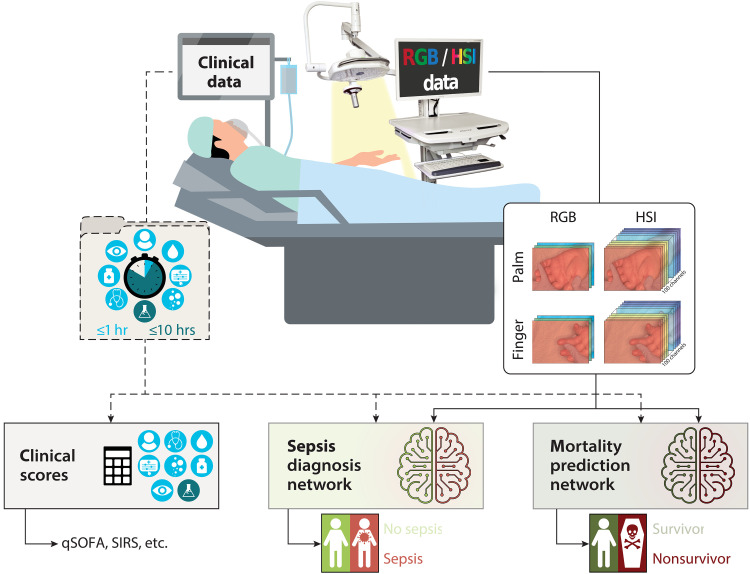
We explore HSI for automated, noninvasive and rapid sepsis diagnosis and mortality prediction. In a prospective study of more than 480 ICU patients, we collected HSI and RGB images of the palm and annular finger and clinical data. Deep learning accurately predicts sepsis and mortality from HSI data, with improved performance when combined with clinical data. Our method outperforms widely used clinical biomarkers and scores such as the qSOFA score and the SIRS criteria. hr, hour.

In summary, despite extensive research efforts, robust biomarkers for early sepsis diagnosis and mortality prediction are still lacking. Here, we close this important gap by presenting the first analysis of deep learning–based HSI analysis for automated, noninvasive, and rapid diagnosis of sepsis and prediction of mortality among ICU patients. On the basis of a prospective study involving more than 480 patients, representing, to the best of our knowledge, the largest medical HSI dataset to date, we investigate the following research questions (compare Fig. 1):

1) Are an automated, noninvasive, and rapid diagnosis of sepsis and prediction of mortality among ICU patients feasible with deep learning–based HSI analysis? What is the optimal measurement site? Do HSI data provide advantages over conventional red-green-blue (RGB) imaging and tissue parameter images (TPIs) derived from HSI in terms of classification performance?

2) Can we further boost the diagnostic and predictive performance by adding structured clinical data?

3) Does our method outperform widely available clinical scores and biomarkers?

## RESULTS

Using the medical device–graded HSI system Tivita 2.0 Surgery Edition (Diaspective Vision, Am Salzhaff, Germany), we collected spectral imaging data from the skin of all patients admitted to the interdisciplinary surgical ICU at the University Hospital Heidelberg. All adult patients admitted between 24 October 2022 and 15 December 2023 were included, resulting in data from 508 patients.

Of these 508 patients, the sepsis status could not be determined for 71 patients. The sepsis diagnosis cohort is thus composed of the remaining 437 patients, of which 129 (30%) were diagnosed with sepsis, while 308 (70%) were not. Among patients with sepsis, the majority (53%) had an abdominal focus, followed by 17% with a respiratory focus, 5% with a skin or soft tissue focus, and 3% with a genitourinary focus. In addition, 8% of the patients with sepsis had multiple foci, while in 14%, the focus of infection remained unknown.

Successful follow-up on 30-day mortality after ICU admission was achieved for 483 of the initial 508 patients. These patients constitute the mortality prediction cohort, of which 68 (14%) died within 30 days of admission. The mortality rate was higher among patients with sepsis and septic shock at the time of admission, at 27% (35 of 129) and 49% (24 of 49), respectively, compared to 6% (18 of 308) for those without sepsis at admission.

The palm and annular finger were chosen as measurement sites because of their easy accessibility and low melanin content. Characteristic tissue spectra for patients with sepsis versus patients without sepsis and survivors versus nonsurvivors are available in fig. S1.

HSI captures tissue reflectance spectra, which are influenced by chromophores like hemoglobin and water within the tissue. Consequently, functional tissue parameter indices can be approximated from HSI data according to the formulas presented in (*29*), including oxygen saturation (the fraction of oxygen-saturated hemoglobin relative to total hemoglobin), perfusion index (a composite measure of perfusion targeting deeper tissue layers), hemoglobin index (indicative of the amount of hemoglobin in the tissue microcirculation), and water index (reflecting the water content in tissue) (*29*, *30*). Furthermore, an integrated RGB sensor captured RGB images alongside the HSI data, allowing for a direct comparison between HSI—a novel imaging modality with enhanced spectral information—and the more widely used, cost-effective, and faster RGB imaging.

### HSI can rapidly and noninvasively diagnose sepsis and predict mortality

**Fig. 2.**
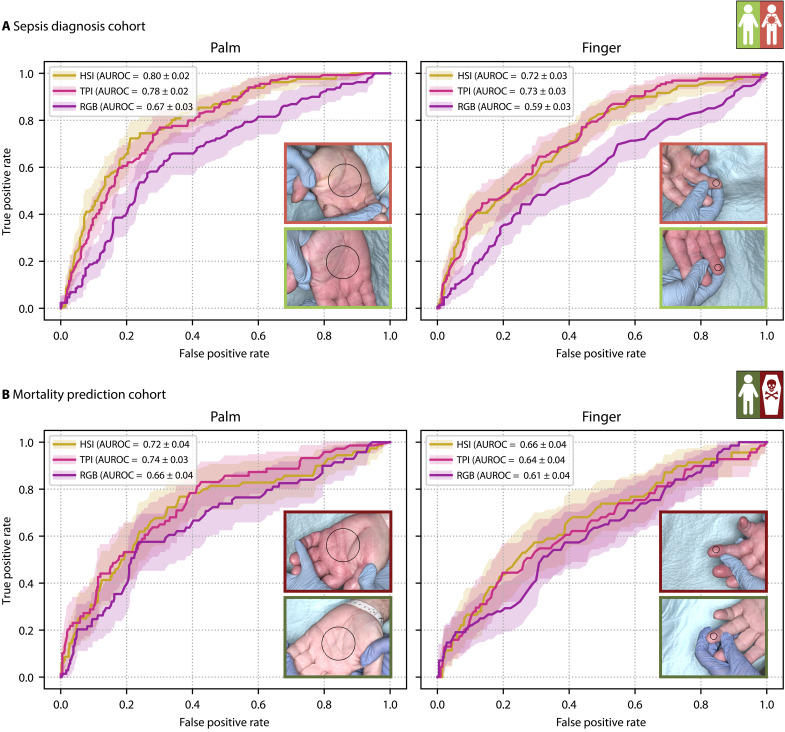
HSI can rapidly and noninvasively diagnose sepsis and predict mortality. Receiver operating characteristics (ROCs) are shown for sepsis diagnosis (**A**) and mortality prediction (**B**) models based on HSI data (gold), stacked tissue parameter images (TPIs; pink), and RGB (violet) data of the palm (left) and annular finger (right).The shaded areas denote the 95% CI across 1000 bootstrap samples, and the mean and standard deviation of the AUROC are reported in the legend. Sample images of a patient with sepsis (light red box) and a patient without sepsis (light green box), as well as a survivor (dark green) and a nonsurvivor (dark red), are included on the bottom right, with the circle denoting the annotated skin region.

As shown in Fig. 2, deep learning–based diagnosis of sepsis from the palm was achievable with an area under the receiver operating characteristic curve (AUROC) of 0.80 [95% confidence interval (CI) [0.76; 0.84]], while the finger measurements yielded an AUROC of 0.72 (95% CI [0.67; 0.78]). Also for mortality prediction, the palm measurement site yielded better classification performance [AUROC of 0.72 (95% CI [0.65; 0.79])] compared to the finger measurements [AUROC of 0.66 (95% CI [0.59; 0.73])]. Combining both measurement sites did not yield substantial performance improvements that would justify the added complexity and effort of acquiring two HSI measurements instead of one (compare fig. S2).

HSI demonstrated superior diagnostic performance compared to conventional RGB imaging, with up to a 23% improvement. The performance of models based on HSI data and those using TPIs derived from HSI data was similar, suggesting that TPIs effectively capture information relevant to sepsis diagnosis and mortality prediction.

### Patients with sepsis and nonsurvivors have decreased palm tissue oxygen saturation at increased tissue water and hemoglobin content

**Fig. 3.**
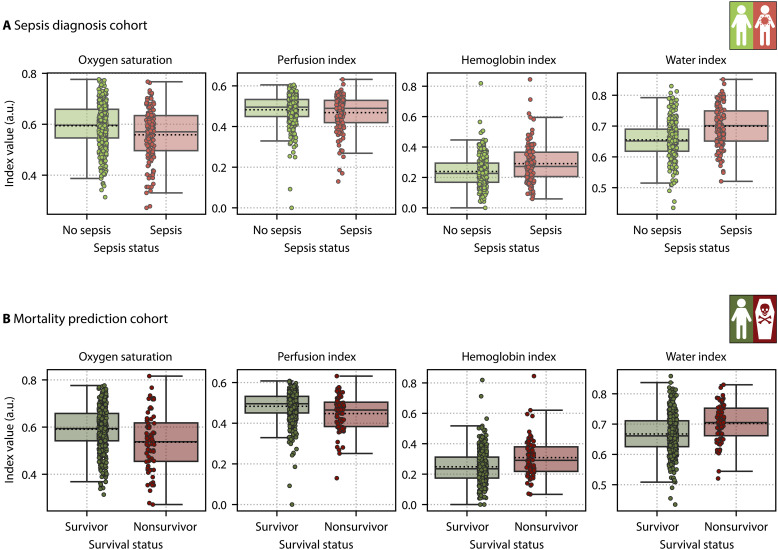
Patients with sepsis and nonsurvivors have significantly lower palm tissue oxygen saturation and higher tissue hemoglobin and water indices. The subfigures show the distribution of the functional parameters oxygen saturation, perfusion index, hemoglobin index, and water index in arbitrary units (a.u.), as derived from HSI palm measurements, for patients with and without sepsis (**A**) and survivors and nonsurvivors (**B**). The boxes denote the quartiles of the distribution with the whiskers extending up to 1.5 times the interquartile range, and the median and mean are drawn as solid and dashed lines, respectively. Each dot represents one patient. Tissue parameter index distributions for the measurement site finger are available in fig. S3.

Distributions of the functional tissue parameter indices oxygen saturation, perfusion index, hemoglobin index, and water index for patients with and without sepsis, as well as survivors and nonsurvivors, are shown in Fig. 3 for the measurement site palm. In patients with sepsis, oxygen saturation was significantly lower compared to patients without sepsis (*P* = 7.1 × 10^−4^), while hemoglobin and water indices were significantly higher (*P* = 6.2 × 10^−5^ and *P* = 4.5 × 10^−10^, respectively). The perfusion index did not show a significant difference (*P* = 1.1 × 10^−1^). In nonsurvivors, the perfusion index and oxygen saturation were significantly lower (*P* = 2.5 × 10^−3^ and *P* = 6.8 × 10^−4^, respectively) compared to survivors, and hemoglobin and water indices were significantly higher (*P* = 6.0 × 10^−4^ and *P* = 7.0 × 10^−5^, respectively). More details on the statistical tests are available in table S1, and the tissue parameter index distributions for the measurement site finger are illustrated in fig. S3.

### Structured clinical data boost the classification performance

Structured clinical data were collected alongside the HSI data, including demographics, vital signs, blood gas analysis measurements, therapy details (usage of organ replacement therapies, ventilation parameters, and dose of administered vasopressors and inotropes), and laboratory results. A total of 45 clinical parameters was recorded, with 33 usually available within 1 hour of admission and the 12 laboratory parameters usually available within 10 hours of admission. Descriptive statistics of the clinical parameters are available in tables S2 and S3.

**Fig. 4.**
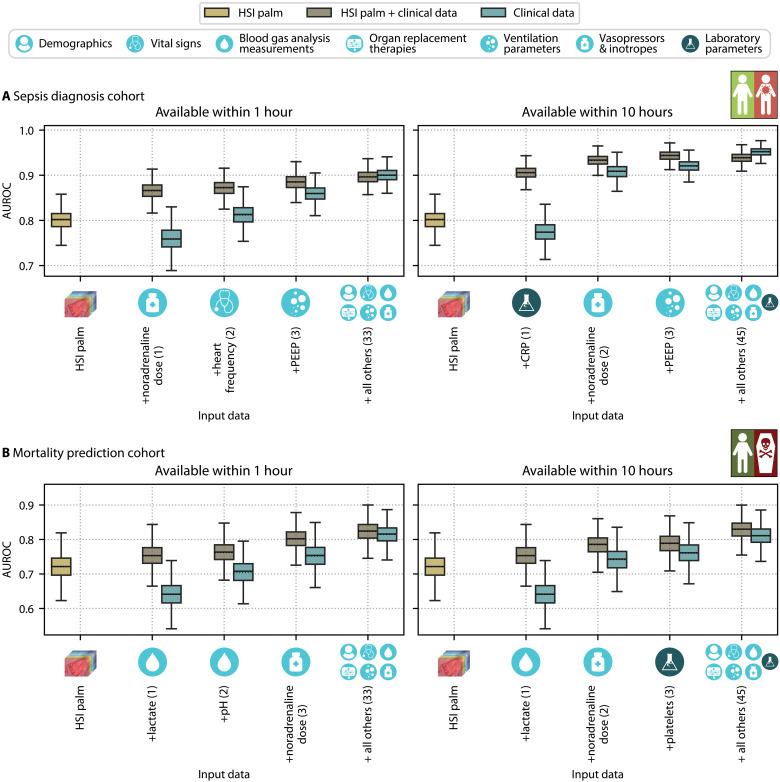
Adding clinical data boosts the sepsis diagnosis and mortality prediction performance. The performance of sepsis diagnosis (**A**) and mortality prediction (**B**) using HSI data of the palm (HSI palm model, gold), a combination of HSI and clinical data (HSI palm + clinical data model, bronze), and clinical data alone (clinical data model, blue) is shown, categorized by data availability within 1 hour (left) and 10 hours (right) from admission to the ICU. Within the subplots, the performance of the HSI palm model is compared to HSI palm + clinical data and clinical data models that incorporate—from left to right—the most important, two most important, three most important, or all clinical data features available within the specified time frame of 1 hour or 10 hours after ICU admission. The number of clinical data features used in the model is indicated in brackets. The ranking of the clinical data features according to feature importance was derived from the clinical data model through RFE (*31*) starting from the complete set of available clinical data at the given time point. Each box plot represents the quartiles of the AUROC distribution across 1000 bootstrap samples, with whiskers extending up to 1.5 times the interquartile range. The median and mean are drawn as solid and dashed lines, respectively.

Incorporating all clinical data available within the first hour of ICU admission alongside HSI data of the palm in the HSI palm + clinical data model improved sepsis diagnosis performance from an AUROC of 0.80 (95% CI [0.76; 0.84]) to 0.90 (95% CI [0.87; 0.92]) (compare Fig. 4). The performance further increased to 0.94 (95% CI [0.92; 0.96]) when laboratory values, available within 10 hours postadmission, were also included. Although a random forest model using comprehensive clinical data alone, referred to as the clinical data model, performed slightly better on the full set of clinical data, combining HSI with clinical data performed substantially better when only limited clinical data were available, a common scenario in emergency settings, outpatient health care, and LMICs. We ranked the importance of clinical data features using recursive feature elimination (RFE) (*31*) with the clinical data model, beginning with the full set of clinical data available within the specified time frame of either 1 hour or 10 hours after ICU admission. An overview of feature importance is presented in fig. S4 for clinical data available within 1 hour and in fig. S5 for clinical data available within 10 hours. As depicted in Fig. 4, sequentially adding clinical data features in the order of their importance revealed that the HSI palm + clinical data model already achieved an AUROC of 0.87 (95% CI [0.83; 0.90]) by combining a single clinical parameter immediately available at the bedside, namely the administered noradrenaline dose, with HSI data.

Combining HSI with clinical data also boosted the mortality prediction performance. The AUROC improved from 0.72 (95% CI [0.65; 0.79]) to 0.82 (95% CI [0.76; 0.88]) when including all clinical data available within the first hour of admission and further to 0.83 (95% CI [0.78; 0.88]) when incorporating all clinical data from the first 10 hours of admission. When clinical data features were sequentially added in order of their importance, the HSI palm + clinical data model consistently outperformed the clinical data model, with the performance advantage being most pronounced when using a smaller number of clinical data features. The three most important clinical data available within 1 hour from admission were lactate, pH, and noradrenaline dose, which achieved an AUROC of 0.80 (95% CI [0.74; 0.85]) in combination with HSI data of the palm.

### Our HSI-based classification models surpass clinical biomarkers and scores for sepsis diagnosis and mortality prediction

We compared our HSI palm + clinical data models using the complete set of clinical data available within 1 hour or 10 hours after ICU admission, as well as our HSI palm models, with commonly used clinical biomarkers and scores for sepsis diagnosis and mortality prediction. Rapidly available bedside scores for sepsis diagnosis include the skin mottling score (SMS) (*32*) and capillary refill time (CRT) (*33*), both of which depend on visual skin assessment. In addition, we compared our sepsis diagnosis models to the quick Sequential Organ Failure Assessment (qSOFA) score (*1*) and the National Early Warning Score (NEWS) (*34*), which are based on vital signs and cognitive function. Among the biomarkers and scores available within 10 hours from admission, we compared our models against the inflammatory biomarkers procalcitonin (PCT) and C-reaction protein (CRP). In addition, we evaluated them against the Systemic Inflammatory Response Syndrome (SIRS) criteria, previously used for sepsis diagnosis (*35*), and the Sequential Organ Failure Assessment (SOFA) score (*1*), which is a key component of the current Sepsis-3 definition.

**Fig. 5.**
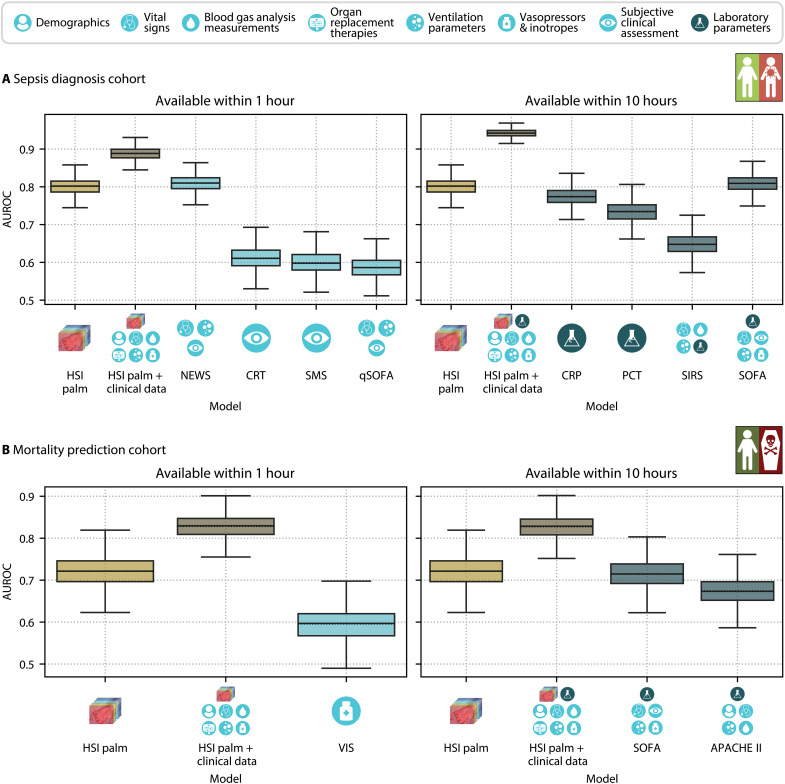
Our HSI + clinical data models outperform widely used clinical biomarkers and scores for sepsis diagnosis and mortality prediction. Comparison of the AUROC for deep learning–based sepsis diagnosis (**A**) and mortality prediction (**B**) using HSI data of the palm (HSI palm model, gold) and a combination of HSI data and the entire set of clinical data available within 1 hour (left) and 10 hours (right) from admission to the ICU (HSI palm + clinical data model, bronze) against clinical biomarkers and scores (blue). For data available within 1 hour of ICU admission, the comparison includes National Early Warning Score (NEWS), capillary refill time (CRT), skin mottling score (SMS), qSOFA score, and VIS. For data available within 10 hours of admission, the comparison includes C-reaction protein (CRP), procalcitonin (PCT), SIRS criteria, SOFA score, and APACHE II score. Each box plot displays the quartiles of the AUROC distribution across 1000 bootstrap samples, with whiskers extending up to 1.5 times the interquartile range. The median and mean are represented by solid and dashed lines, respectively.

Commonly used clinical biomarkers and scores for assessing disease severity and risk of mortality include the vasoactive inotropic score (VIS) (*36*), the SOFA score (*1*), and the Acute Physiology and Chronic Health Evaluation (APACHE) II score (*37*). The VIS, which quantifies hemodynamic support on the basis of vasopressor and inotrope doses, is available within 1 hour of admission. Within 10 hours, the SOFA score, assessing organ dysfunction, and the APACHE II score, measuring disease severity, are available. These scores are derived from various vital signs, laboratory parameters, and patient and therapy characteristics, typically using the most abnormal readings within the past 24 hours. To compare our HSI + clinical data models with clinical scores, we used modified versions of the SOFA and APACHE II scores, which were based on the most current data rather than the worst values over 24 hours, ensuring that values were available on the day of admission. As shown in Fig. 5, our HSI + clinical data models outperformed all clinical biomarkers and scores for both sepsis diagnosis and mortality prediction.

## DISCUSSION

In this study, we addressed the critical need for reliable biomarkers to identify patients with sepsis and those at high risk of mortality. We demonstrate that the automated, noninvasive, and rapid diagnosis of sepsis and prediction of mortality among ICU patients is feasible using deep learning–based HSI analysis. Based on the—to the best of our knowledge—largest HSI patient cohort to date, we derived the following key findings:

1) HSI-based prediction: Both sepsis and mortality can be predicted from HSI data with high accuracy using deep learning. Patients with sepsis and nonsurvivors have significantly lower tissue oxygen saturation and higher tissue hemoglobin and water content than patients without sepsis and nonsurvivors. Predictions from HSI measurements of the palm are superior to those from the annular finger.

2) Combination with structured clinical data: Incorporating structured clinical data enhances classification performance, yielding AUROCs of up to 0.94 and 0.83 for sepsis diagnosis and mortality prediction, respectively.

3) Comparison to clinical biomarkers and scores: Our HSI + clinical data models outperform widely available clinical biomarkers and scores that were suggested for sepsis diagnosis and mortality prediction.

### Strengths and limitations of our HSI-based prediction

We believe that the primary strengths of our HSI-based sepsis diagnosis and mortality prediction are its objectivity, noninvasiveness, cost-effectiveness, and speed, as predictions can be obtained from a single HSI cube acquired at the bedside within seconds. Given these advantages, our method could be applied as a screening tool across the entire cohort of critically ill ICU patients, enabling the objective and timely identification of those at high risk for sepsis and mortality. This, in turn, could support the rapid initiation of further diagnostic evaluations and therapeutic interventions. In addition, HSI systems enable mobile measurements and could thus be performed in various hospital wards, such as the emergency department, or even in ambulances. Although HSI systems are not yet widely used clinically, they have evolved rapidly over the past two decades from custom research prototypes to medically certified systems, like the one used in this study (*38*). Manufacturers such as imec (Leuven, Belgium) and HAIC (Hanover, Germany) are currently focusing on developing more compact, real-time HSI devices and scaling production to achieve high-volume, low-cost availability.

We acknowledge that our classification models based on HSI data alone may not be sufficiently accurate as a stand-alone diagnostic and prognostic tool. However, we believe that HSI has high potential as a prescreening tool to identify patients for which time-consuming and costly tests (e.g., laboratory measurements) and extensive monitoring should be performed. This is particularly advantageous in resource-limited settings, such as LMICs, where approximately half of critical care interventions are delivered outside of the ICU (*39*), and in situations requiring immediate decisions, such as emergency treatment.

We showed that substantial performance improvements are possible by integrating a few clinical parameters available at the bedside. For instance, including the administered noradrenaline dose as an additional input improved the AUROC for sepsis prediction from 0.80 (95% CI [0.76; 0.84]) to 0.87 (95% CI [0.83; 0.90]). We want to emphasize, however, that incorporating clinical data may introduce biases and limit generalizability. For example, treatment choices such as the administered noradrenaline dose depend on the implementation of clinical guidelines, which are subject to variation over time and across health systems.

While we showed that the HSI + clinical data models largely outperformed widely used clinical biomarkers and scores and achieved excellent sepsis diagnosis and mortality prediction performance, another limitation of models that require clinical data is that prospectively collecting clinical data requires substantial labor. We decided against the less labor-intensive export of EHR data, as several clinical parameters are not reliably recorded in the EHR, which would lead to inaccuracies. In addition, many clinical parameters, such as vital signs and ventilation parameters, were stored in the EHR at a poor temporal resolution, failing to accurately reflect the patient’s status at the time of the HSI measurement. Given the labor-intensive collection of clinical data, minimizing the number of clinical parameters required for prediction is advantageous. Our results demonstrate that our HSI + clinical data models outperform clinical data models for both sepsis diagnosis and mortality prediction when only few clinical parameters are available.

### Comparison to the state of the art

In 2021, we pioneered machine learning–based sepsis diagnosis using HSI. While our framework was able to differentiate sepsis patients from a control group comprising healthy volunteers and patients undergoing pancreatic surgery with high performance [AUROC of 0.91 (95% CI [0.85; 0.96])], we identified several potential sources of bias, including differences in age, comorbidities, and therapies between patients with and without sepsis, as well as imaging-related factors such as variations in hardware and acquisition protocols. We concluded that these biases may have inflated the algorithm’s performance and could limit its generalizability to real-world clinical settings, such as automated sepsis diagnosis in critically ill ICU patients (*25*). In the spirit of good scientific practice, we therefore never submitted our work to peer review but chose to design this new prospective study for automated sepsis diagnosis and mortality prediction specifically in ICU patients. With our new carefully designed dataset, we achieved lower performance compared to our original work despite using the same machine learning framework. Moreover, the performance of our model trained on the potentially biased dataset dropped markedly to an AUROC of 0.73 (95% CI [0.69; 0.78]) when applied to the new data. These two facts together underscore our hypothesis that prior HSI studies (*23*, *25*–*28*), which compared patients with sepsis with healthy volunteers or selectively chosen cohorts, have limited relevance for accurately assessing the practical feasibility of automated sepsis diagnosis in real-world clinical settings.

### Future work

A key limitation of our study is that all data were collected from a single surgical ICU in Germany. As expected for this setting, most patients with sepsis had an abdominal focus, while other infection sites, such as respiratory (17%) and genitourinary (3%), were less common. Furthermore, the management of critically ill patients varies across clinical sites. While some sites manage critically ill patients in emergency wards before transferring them to the ICU, at our site (Heidelberg University Hospital, Germany), critically ill patients—whether newly admitted from the emergency ward or those with postoperative complications from the general ward—are immediately transferred to the ICU. As a result, patients with sepsis in our ICU cohort may be in the earlier stages of the disease compared to those in ICU cohorts at other clinical sites. Given these variations in ICU populations, external validation is necessary to assess the generalizability of our models across diverse ICU settings and clinical sites with different patient populations.

Given the key strengths of our HSI-based classification models, which enable a rapid, noninvasive, cost-effective, and mobile assessment of sepsis diagnosis and mortality, investigating their performance in resource-constrained and time-critical settings, such as ambulances, emergency wards, and LMICs, is a promising future direction. Furthermore, it would be valuable to explore whether HSI, in addition to detecting patients with sepsis, has the potential to identify individuals earlier in the disease progression, hours or even days before the onset of organ dysfunction. In addition, because an estimated 40 of sepsis cases in 2017 occurred in children under 5 years old (*2*), expanding the cohort to include infants is of interest.

Furthermore, while our observational study identified potential use cases and demonstrated high accuracy in automated HSI-based sepsis diagnosis and mortality prediction, future interventional studies are needed to assess the clinical effectiveness of implementing an automated sepsis and mortality alert system based on our algorithms in the management of critically ill patients. Such studies should compare the system to the standard of care, evaluating its impact on key clinical outcomes, including reductions in mortality, morbidity, and length of hospital stay. To date, only few studies have systematically investigated the clinical effectiveness of automated sepsis and mortality alert systems (*40*).

While we consider our single time-point measurements advantageous for enabling immediate diagnosis and low resource requirements, future studies collecting longitudinal HSI data could expand the potential of HSI. Longitudinal data could improve the understanding of disease progression by identifying features associated with clinical improvement or deterioration.

Besides using HSI for disease diagnosis and prognosis, HSI holds the potential to support novel therapeutic strategies by continuously assessing tissue microcirculation, evaluating treatment effects, and guiding interventions. In the example of shock therapy, the current therapeutic target is macrohemodynamic stabilization (e.g., maintaining normative arterial blood pressure). However, critically ill patients, particularly those with sepsis, often experience a loss of hemodynamic coherence, resulting in dissociation between macro- and microcirculation (*41*). While no other widely available clinical data can capture the spatial distribution of tissue microcirculation, HSI offers a unique opportunity for real-time monitoring. While our primary objective was to identify patients with sepsis and those at risk of mortality, we performed an initial experiment investigating whether differentiation between sepsis and septic shock is feasible with HSI. As demonstrated in fig. S6, a shock classification model achieved AUROCs of 0.66 (95% CI [0.57; 0.75]) at the palm and 0.62 (95% CI [0.51; 0.71]) at the fingers. Future research should further investigate the role of HSI in guiding therapy, not only in shock but also in patients with sepsis and those at high risk of mortality, to assess its potential for improving patient outcomes.

### Conclusions

In this study, we addressed the critical need for reliable biomarkers to identify patients with sepsis and those at high risk of mortality. We investigated the potential of HSI for sepsis diagnosis and mortality prediction in ICU patients on the basis of a prospective study with the largest HSI patient cohort to date, involving more than 480 patients. Our proposed HSI models demonstrated high predictive performance, which improved further when combined with minimal clinical data. They outperformed widely used clinical biomarkers and scores. Key strengths of HSI-based predictions include their rapid, noninvasive, cost-effective, and mobile measurements, making them promising candidates for various clinical settings, including resource-limited scenarios (e.g., LMICs) and time-critical situations (e.g., ambulances and emergency wards). Beyond their proven benefit in sepsis diagnosis and mortality prediction, HSI-based microcirculatory monitoring could also offer novel therapeutic strategies and enhance the understanding of disease progression. Our code and pretrained models will be made publicly available in our GitHub repository (https://github.com/IMSY-DKFZ/htc) and Zenodo (https://doi.org/10.5281/zenodo.6577614) (*42*).

## MATERIALS AND METHODS

### Experimental design

In this prospective observational study, we collected HSI data and corresponding RGB images from the skin of patients admitted to the interdisciplinary surgical ICU at the University Hospital Heidelberg (Germany). All adult patients admitted between 24 October 2022 and 15 December 2023 were included. The study was conducted in accordance with the ethical standards laid down in the 1964 Declaration of Helsinki and its later amendments. The protocol was approved by the Ethics Committee of the Medical Faculty of Heidelberg University, Germany (study reference number: S-288/2022) and registered with the German Clinical Trials Register (study identifier: DRKS00029709) before the commencement of recruitment. The palm and annular finger were selected as measurement sites for their easy accessibility and low melanin content, with the hand chosen to ensure that it was not used for intra-arterial cannulas or intravascular access. Structured clinical data were collected alongside the HSI data, including demographics, vital signs, blood gas analysis measurements, therapy details (usage of organ replacement therapies, ventilation parameters, and dose of administered vasopressors and inotropes), and laboratory results. In total, 45 clinical parameters were recorded, with 33 usually available within 1 hour and 45 parameters usually available within 10 hours of admission. Tables S2 and S3 provide descriptive statistics of the clinical data.

### Hyperspectral image acquisition

The camera system used was the medical device–graded TIVITA 2.0 Surgery Edition (Diaspective Vision GmbH, Am Salzhaff, Germany). It features a push-broom design with a spectral resolution of ~5 nm, covering 100 spectral channels in the range of 500 to 1000 nm. The resulting HSI cubes have dimensions of 640 by 480 by 100 (width × height × number of spectral channels). The imaged area is approximately 16 by 11.5, with an imaging distance of about 50 cm, maintained by an integrated distance calibration system. Image acquisition takes ~7 s.

The system includes both HSI and RGB sensors, providing simultaneous RGB images with dimensions of 640 by 480 by 3 (width × height × number of channels). TPIs, such as oxygen saturation, perfusion index, hemoglobin index, and water index, are estimated from the HSI data according to the formulas published in (*29*).

During image acquisition, window blinds were lowered, and all light sources other than the integrated light-emitting diode unit were turned off. The hands of patients were supported by the examiner to prevent motion artifacts and ensure more uniform hand positioning, with a consistent background used across all images.

### Hyperspectral image annotation

Despite using a uniform background and standardizing hand positioning as much as possible, images might still include elements such as dressings, wounds, wires, tubes, or parts of the examiner’s gloved hand. To mitigate potential confounding from these elements, our analysis was performed on annotated skin areas. We chose circular annotations to consistently capture the same measurement sites across patients, regardless of hand rotation in the imaging plane. According to our annotation guidelines, the selected annotation radii were 100 pixels for the palm and 20 pixels for the ring finger. Finger annotations were centered on the fingertip, and palm annotations were centered on the palm of the hand, defined as the area enclosed by the wrist, the metacarpophalangeal joints, and the thumb basal joint.

### Sepsis and outcome labels

Diagnosis of sepsis was based on the Sepsis-3 criteria, which define it as acute, life-threatening organ dysfunction resulting from a suspected or confirmed infection (*1*). Organ dysfunction was evaluated using the SOFA score, with an acute increase of at least two points indicating sepsis. Differentiating between organ failure caused by sepsis and that resulting from nonseptic inflammation can be challenging, particularly in a surgical ICU setting following surgical trauma. To maintain label quality and avoid ambiguity in such cases, we introduced a third label, “unsure,” alongside the labels “sepsis” and “no sepsis.” For each patient, the sepsis status was independently assessed by two expert anesthetists. Disagreements between the two anesthetists were resolved by a third, more senior anesthetist (the head of the department for anesthesia and intensive care). Mortality was assessed through a follow-up conducted 30 days after the patient’s inclusion.

### Data preprocessing

Following calibration of HSI cubes using white and dark reference cubes, ${𝓁}^{1}$-normalization was applied across the spectral channels. The tissue parameter index images such as oxygen saturation, perfusion index, hemoglobin index, and water index were computed from the HSI cubes using the formulas presented in (*29*) and subsequently stacked to form a TPI cube, with dimensions of 640 by 480 by 4 (width × height × number of channels). HSI, TPI, and RGB cubes were cropped to a square that tightly encompassed the circular annotation, with pixels outside the annotated area set to zero. The cropped cubes were rescaled to dimensions of 224 by 224 by 100, 224 by 224 by 4, and 224 by 224 by 3, respectively (width × height × number of spectral channels). To enable a direct comparison between classification models using HSI data from both palm and finger measurement sites versus those using only palm or finger data, cropped HSI cubes from both sites for the same patient were stacked along the spectral dimension. This resulted in cubes with dimensions of 224 by 224 by 200, which were used as input for the HSI palm + finger model.

The missingness in the clinical parameters was low, averaging at 1.6%. Missing values were imputed with −1.

### Classification models

We developed deep learning classifiers for automated sepsis diagnosis and mortality prediction using solely HSI data (HSI model), TPI cubes (TPI model), RGB data (RGB model), and clinical data (clinical data model), as well as a multimodal approach combining HSI with clinical data (HSI + clinical data model). The HSI, TPI, and RGB models are based on a convolutional neural network (CNN) architecture. CNNs were chosen for their widespread use in medical HSI classification and their advantages over traditional machine learning methods, including higher model accuracy and efficient computation through shared weights and hardware optimizations (*43*). Using standardized architectures with pretrained weights allows for faster convergence and often yields better performance than training CNNs from scratch, particularly when working with small medical datasets (*44*). To this end, our HSI, TPI, and RGB models are composed of a ResNet14d (*45*, *46*) architecture with pretrained ImageNet weights.

The HSI + clinical data model consists of two submodels: The HSI data are processed equivalently to the HSI model by a ResNet14d model with pretrained ImageNet weights up to the bottleneck layer. The clinical data are handled by a submodel comprising two fully connected blocks. Each block includes a linear, batch normalization, exponential linear unit activation, and dropout layer. The linear layer in the first block has a size of 50, while a size of 30 is used in the second block. The two blocks are followed by a linear head with a size of 10, matching the bottleneck layer size of the HSI submodel. After batch normalization of both bottleneck layers, the bottleneck features are concatenated and fed into another fully connected block, followed by the final classification head.

For the HSI, TPI, RGB, and HSI + clinical data models, cross-entropy loss was used during training. The same hyperparameter settings were applied across all deep learning models. Data augmentations included rotations up to ±180 and horizontal and vertical flipping, each with a probability of 0.5. The AdamW optimizer (*47*) and an exponential learning rate schedule were used (initial learning rate: 0.001; decay rate γ: 0.99; Adam decay rate β_1_: 0.9; Adam decay rate β_2_: 0.999). To regularize the network, a weight decay of 0.001 was applied. Training was conducted for 10 epochs, each consisting of 500 images, with stochastic weight averaging (*48*) applied over the past two epochs. A batch size of 32 images was used, and underrepresented classes were oversampled in each batch to ensure equal class distribution.

For the clinical data model, we used a random forest classifier composed of 100 trees, as it is widely used in sepsis diagnosis from EHR data (*49*). The implementation from sklearn (*50*) was used with default settings, except that balanced class weighting was enabled to adjust the weights inversely proportional to the class frequencies in the training data.

### Training and validation setup

The same training and validation setup was used across all trained models. Given the limited dataset size, we decided against a single hold-out test set for model validation. Instead, we implemented a nested cross-validation scheme, providing more robust performance estimation based on the entire dataset (*51*). The number of outer and inner folds was set to five.

To further stabilize the performance of the trained networks, each run was repeated three times with different random seed settings, altering the initialization of workers and the order in which images were seen during training. On the validation sets, ensembling was performed over these three repetitions. For the test data, the networks from all five folds and three repetitions each (a total of 15 networks) were ensembled by averaging the predictions (logits).

Following the recommendations in (*52*), the model performances were validated using the receiver operating characteristic (ROC) curve and AUROC. To compute CIs that reflect sampling variability, bootstrap sampling was repeated 1000 times for each test set *T*, with ∣T∣ samples randomly drawn with replacement for each bootstrap.

### Feature importance of clinical data

The feature importance of the clinical data was determined using RFE (*31*) from the clinical data models. RFE was adapted to the fivefold cross-validation setup of our inner folds by averaging the feature importance across inner folds before eliminating the least important feature from the set of input features. This process was performed independently on all five outer folds.

### Statistical analysis

Statistical tests were conducted to identify significant differences in functional tissue parameter values between patients with and without sepsis, as well as between survivors and nonsurvivors, resulting in four tests for each group. Two-sided Welch’s *t* test (*53*) was applied, with an overall significance level of 0.05 for each group of tests. To prevent the accumulation of alpha errors because of multiple testing, Bonferroni correction (*54*) was applied, setting the significance level at 0.0125 per test.

### Use of artificial intelligence

During the preparation of this manuscript, we used a large language model (GPT-3.5) for language improvements. We used the following prompt: “Please revise the following text to improve its clarity, conciseness, and scientific tone. Use academic language and follow the Science Advances style guidelines. Avoid overly complex phrasing, but ensure precision and objectivity. Do not change the meaning or content of the original text. Text: [...]”.
