# Supplementary material for: AI-powered skin spectral imaging enables instant sepsis diagnosis and outcome prediction in critically ill patients
Source: Sci Adv. 2025 Jul 18;11(29):eadw1968. doi: 10.1126/sciadv.adw1968 (PMC12273750; doi:10.1126/sciadv.adw1968)
Supplement: Supplementary file 1 — Figs. S1 to S6 Tables S1 to S3 [file sciadv.adw1968_sm.pdf]

Science Advances
AAAS

Supplementary Materials for

AI-powered skin spectral imaging enables instant sepsis diagnosis and outcome prediction in critically ill patients

Silvia Seidlitz *et al.*

Corresponding author: Silvia Seidlitz, s.seidlitz@dkfz-heidelberg.de; Lena Maier-Hein, l.maier-hein@dkfz-heidelberg.de; Maximilian Dietrich, maximilian.dietrich@med.uni-heidelberg.de

Sci. Adv. **11**, eadw1968 (2025)
DOI: 10.1126/sciadv.adw1968

This PDF file includes:

Figs. S1 to S6
Tables S1 to S3

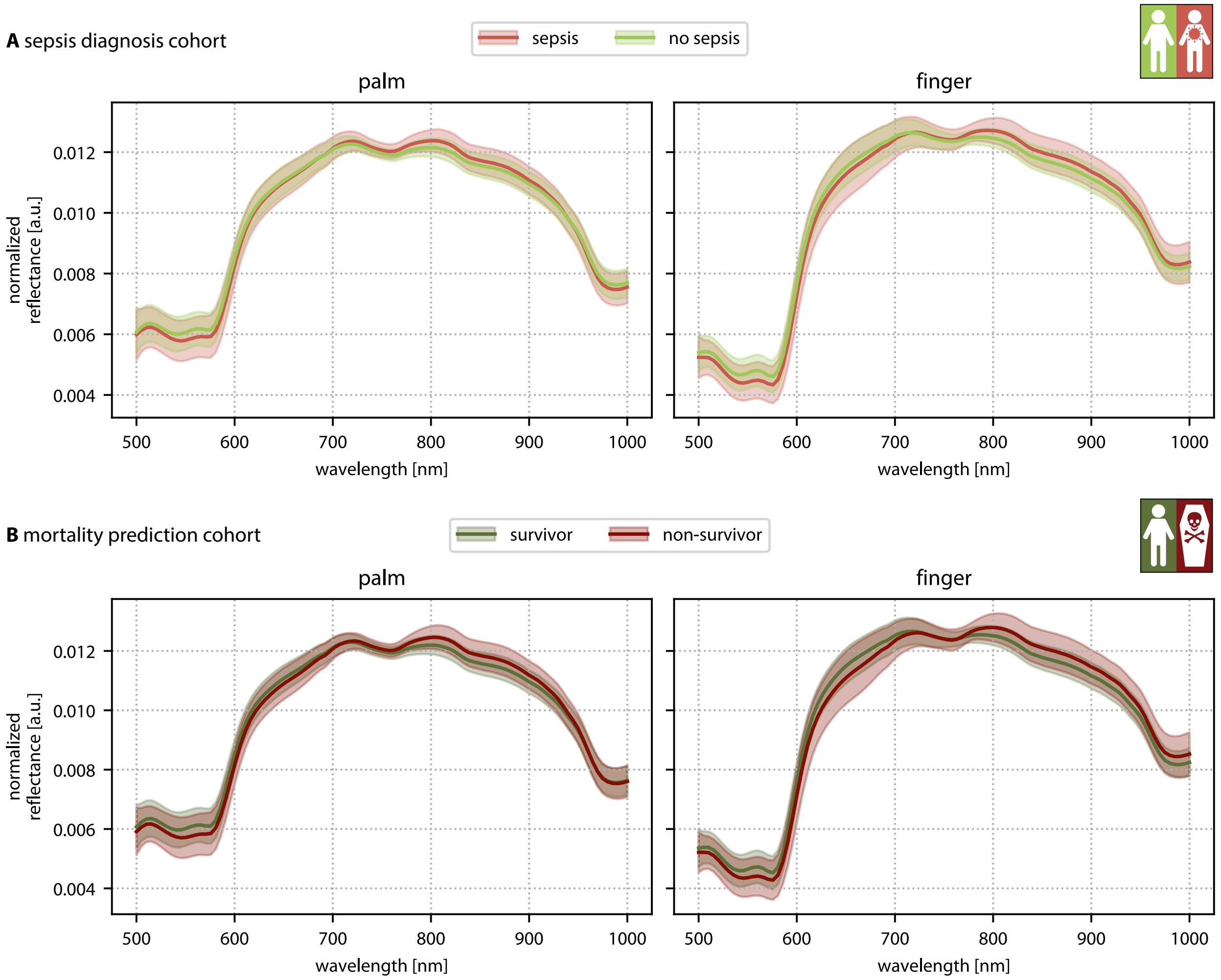

Figure S1: **Characteristic spectra for septic vs. non-septic patients (A) and survivors vs. non-survivors (B).** The plots display the average ℓ^1-normalised spectra across patients (solid lines), with shaded areas indicating one standard deviation, for the measurement sites palm (left) and finger (right).

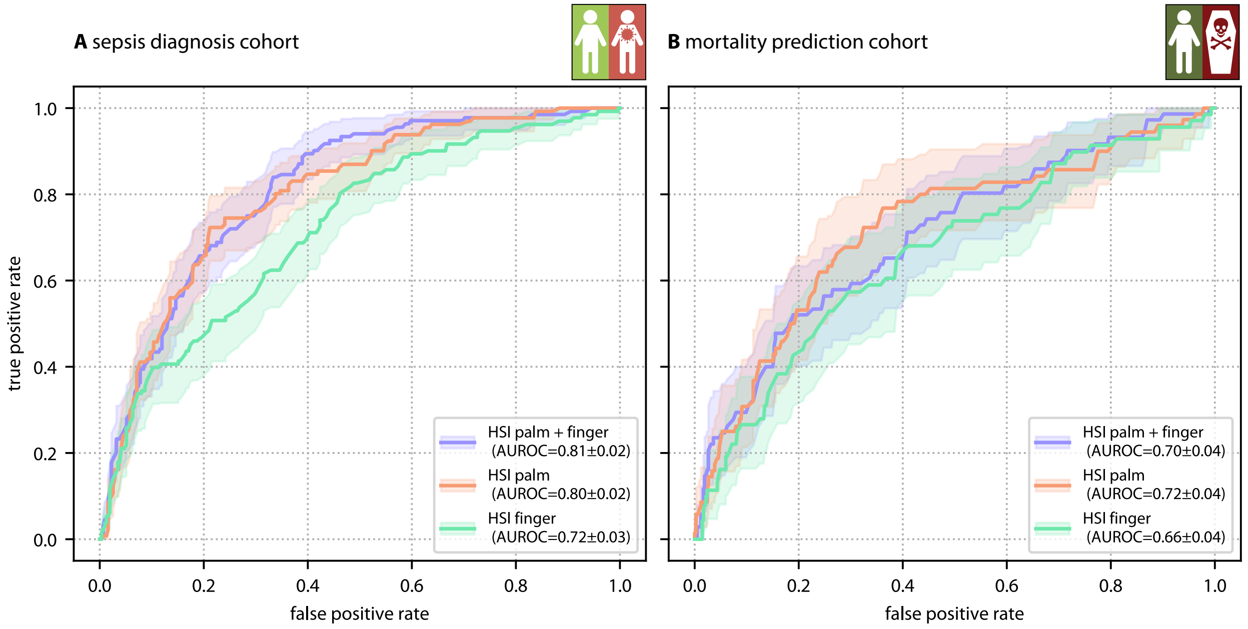

Figure S2: **Combining palm and finger measurements does not provide a substantial advantage over using palm measurements alone.** Receiver operating characteristics are shown for deep learning-based sepsis diagnosis (**A**) and mortality prediction (**B**) using hyperspectral imaging (HSI) data from the palm (`HSI palm` model), the finger (`HSI finger` model), and a combination of both (`HSI palm + finger` model). Shaded areas represent the 95 % confidence interval across 1000 bootstrap samples, with the mean and standard deviation of the area under the receiver operating characteristic curve (AUROC) reported in the legend.

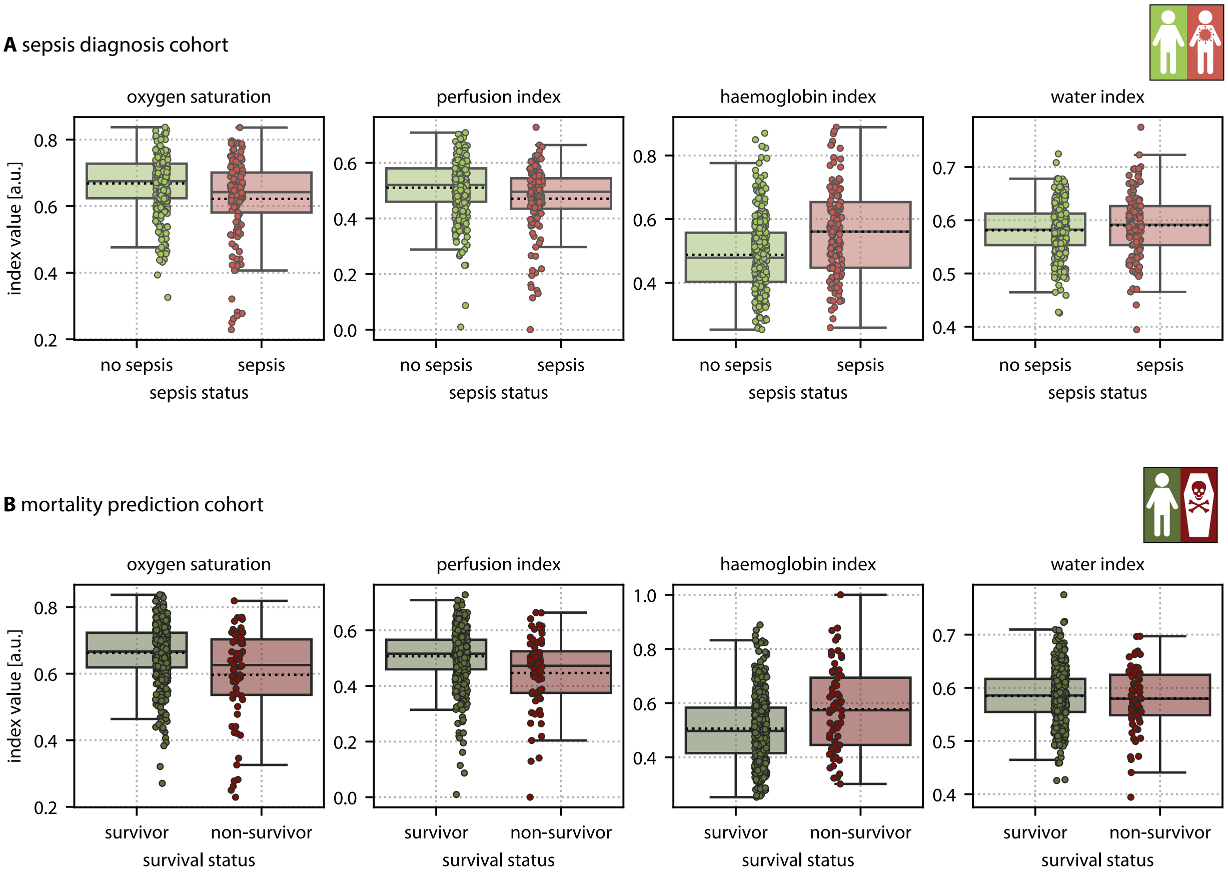

Figure S3: **Septic patients and non-survivors possess significantly lower finger tissue oxygen saturation, and higher tissue haemoglobin and perfusion index.** The subfigures show the distribution of the functional parameters oxygen saturation, perfusion index, haemoglobin index and water index, derived from hyperspectral imaging finger measurements, for septic and non-septic patients (**A**), and survivors and non-survivors (**B**). The boxes denote the quartiles of the distribution with the whiskers extending up to 1.5 times the interquartile range, and the median and mean drawn as solid and dashed lines, respectively. Each dot represents one patient.

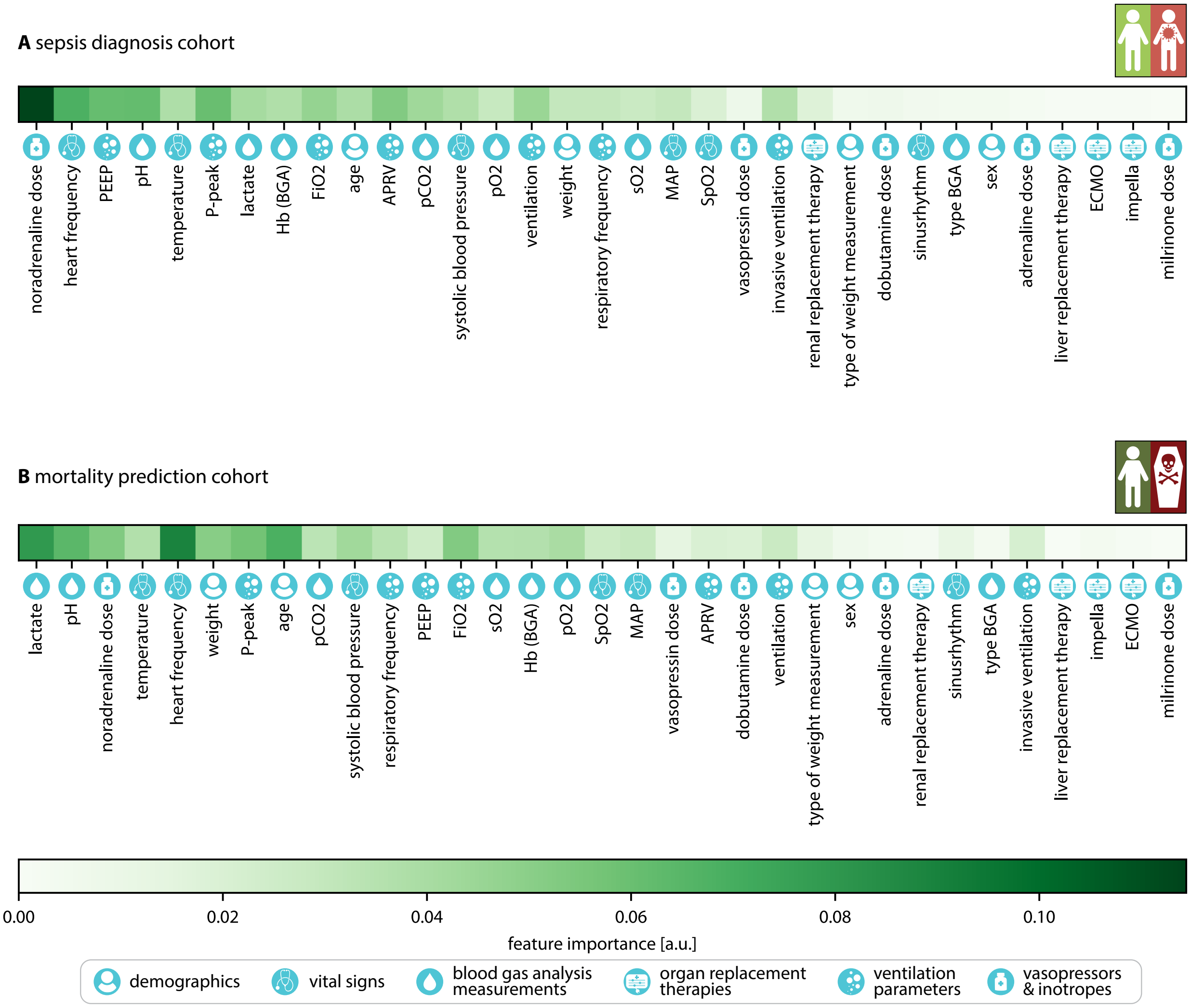

Figure S4: **Feature importance of clinical data available within one hour of intensive care unit admission for sepsis diagnosis (A) and mortality prediction (B) using the `clinical data` model.** Colors represent feature importance based on the reduction in Gini importance when a specific feature is used for data splitting within a decision tree node. Clinical data features are ordered by importance as determined through recursive feature elimination (*31*), from most important (left) to least important (right).

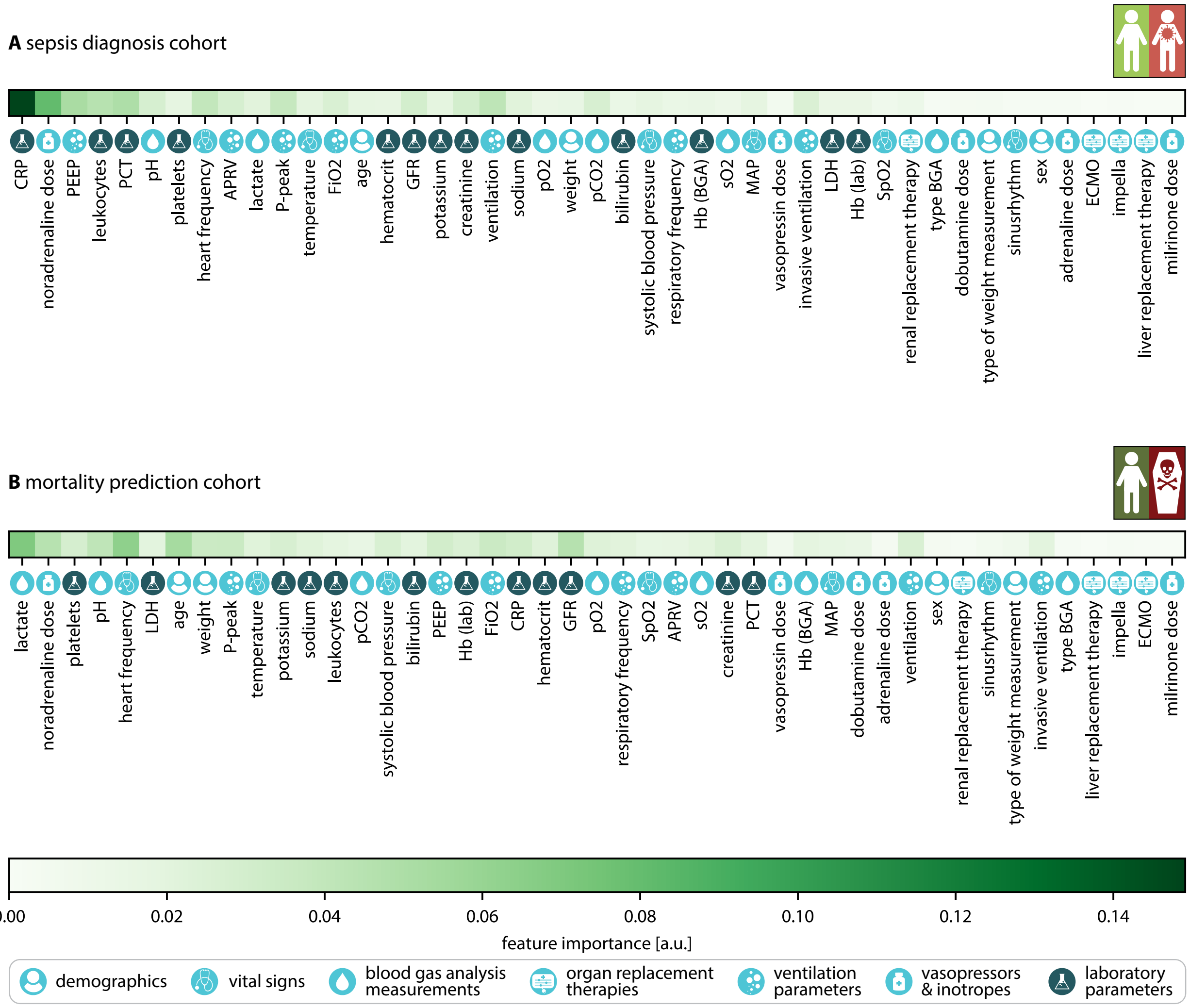

Figure S5: **Feature importance of clinical data available within ten hours of intensive care unit admission for sepsis diagnosis (A) and mortality prediction (B) using the `clinical data` model.** Colors represent feature importance based on the reduction in Gini importance when a specific feature is used for data splitting within a decision tree node. Clinical data features are ordered by importance as determined through recursive feature elimination (*31*), from most important (left) to least important (right).

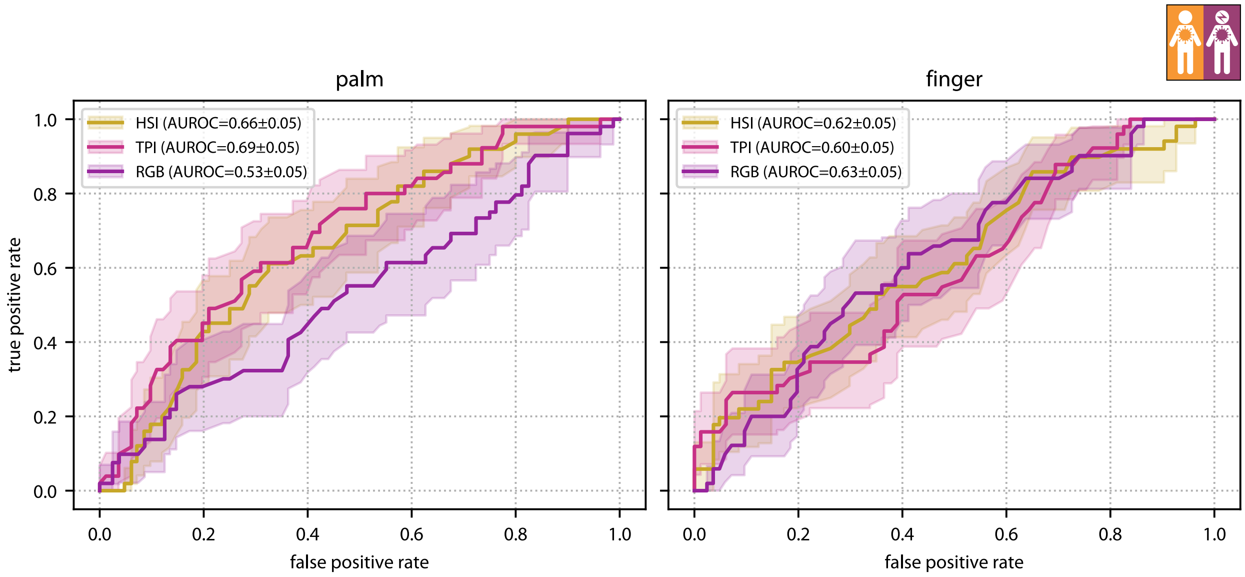

Figure S6: **Hyperspectral imaging (HSI) can rapidly and non-invasively differentiate between sepsis and septic shock patients.** Among our 129 septic patients, 49 (38 %) experienced septic shock. Receiver operating characteristics are shown for classification models based on HSI data (gold), stacked tissue parameter images (TPI, pink) and red-green-blue (RGB, violet) data of the palm (left) and annular finger (right). The shaded areas denote the 95 % confidence interval across 1000 bootstrap samples, and mean and standard deviation of the area under the receiver operating characteristic curve (AUROC) are reported in the legend.

Table S1: Two-sided Welch's t-tests (*53*) were performed to determine significant differences in functional tissue parameter values of palm and finger measurements based on sepsis status and survival status. A summary of p-values, degrees of freedom (DOF), t-statistic and 95 % confidence interval (CI) is provided.

site	target	functional parameter	p-value	DOF	t-statistic	95 % CI
palm	sepsis status	oxygen saturation	$7.1 \cdot 10^{-4}$	208	-3.44	$[-0.06; -0.02]$
palm	sepsis status	perfusion index	$1.1 \cdot 10^{-1}$	205	-1.63	$[-0.03; 0.00]$
palm	sepsis status	haemoglobin index	$6.2 \cdot 10^{-5}$	198	4.09	$[0.03; 0.08]$
palm	sepsis status	water index	$4.5 \cdot 10^{-10}$	222	6.53	$[0.03; 0.06]$
palm	survival status	oxygen saturation	$6.8 \cdot 10^{-4}$	79	-3.54	$[-0.09; -0.02]$
palm	survival status	perfusion index	$2.5 \cdot 10^{-3}$	82	-3.12	$[-0.06; -0.01]$
palm	survival status	haemoglobin index	$6.0 \cdot 10^{-4}$	81	3.57	$[0.03; 0.09]$
palm	survival status	water index	$7.0 \cdot 10^{-5}$	93	4.16	$[0.02; 0.05]$
finger	sepsis status	oxygen saturation	$1.4 \cdot 10^{-4}$	176	-3.89	$[-0.07; -0.02]$
finger	sepsis status	perfusion index	$1.5 \cdot 10^{-3}$	196	-3.22	$[-0.06; -0.02]$
finger	sepsis status	haemoglobin index	$4.4 \cdot 10^{-7}$	205	5.22	$[0.05; 0.10]$
finger	sepsis status	water index	$1.2 \cdot 10^{-1}$	194	1.56	$[-0.00; 0.02]$
finger	survival status	oxygen saturation	$3.7 \cdot 10^{-4}$	75	-3.73	$[-0.10; -0.03]$
finger	survival status	perfusion index	$5.4 \cdot 10^{-4}$	79	-3.61	$[-0.09; -0.03]$
finger	survival status	haemoglobin index	$4.6 \cdot 10^{-4}$	81	3.65	$[0.03; 0.11]$
finger	survival status	water index	$5.6 \cdot 10^{-1}$	84	-0.59	$[-0.02; 0.01]$

Table S2: Descriptive statistics are provided for patients with and without sepsis, as well as for survivors and non-survivors. This includes clinical data available within the first hour of admission to the intensive care unit, such as demographics, vital signs, blood gas analysis (BGA) measurements, use of organ replacement therapies, ventilation parameters, and dose of administered vasopressors and inotropes. For ratio-scaled parameters, means are presented with standard deviation (SD) in brackets. For nominal-scaled parameters, the number of patients per category is listed, while for boolean therapy parameters, the percentage of patients receiving the treatment is provided. Abbreviations denote the mean arterial pressure (MAP), pulse oxymetrical oxygen saturation (SpO2), carbon dioxide partial pressure (pCO2), oxygen partial pressure (pO2), oxygen saturation (sO2), haemoglobin concentration (Hb), extracorporeal membrane oxygenation (ECMO), airway pressure release ventilation (APRV), fraction of inspired oxygen (FiO2), positive endexpiratory pressure (PEEP), and peak inspiratory pressure (P-peak).

attribute	no sepsis	sepsis	non survivor	survivor
number of subjects	308	129	68	415
		**demographics**		
age	$6.2 \cdot 10^1$ ($1.5 \cdot 10^1$)	$6.6 \cdot 10^1$ ($1.4 \cdot 10^1$)	$6.9 \cdot 10^1$ ($1.5 \cdot 10^1$)	$6.3 \cdot 10^1$ ($1.4 \cdot 10^1$)
sex	220 male<br>88 female	90 male<br>39 female	41 male<br>27 female	299 male<br>116 female
weight [kg]	$8.2 \cdot 10^1$ ($2.0 \cdot 10^1$)	$8.2 \cdot 10^1$ ($2.6 \cdot 10^1$)	$7.5 \cdot 10^1$ ($2.3 \cdot 10^1$)	$8.2 \cdot 10^1$ ($2.1 \cdot 10^1$)
type of weight measurement	245 estimated<br>53 measured	100 estimated<br>16 measured	52 estimated<br>8 measured	331 estimated<br>68 measured
		**vital signs**		
heart frequency [bpm]	$8.2 \cdot 10^1$ ($1.7 \cdot 10^1$)	$9.9 \cdot 10^1$ ($2.1 \cdot 10^1$)	$9.8 \cdot 10^1$ ($2.4 \cdot 10^1$)	$8.6 \cdot 10^1$ ($1.9 \cdot 10^1$)
sinusrhythm [%]	79	74	60	78
MAP [mmHg]	$8.1 \cdot 10^1$ ($1.4 \cdot 10^1$)	$7.6 \cdot 10^1$ ($1.3 \cdot 10^1$)	$7.7 \cdot 10^1$ ($1.3 \cdot 10^1$)	$8.0 \cdot 10^1$ ($1.4 \cdot 10^1$)
systolic blood pressure	$1.2 \cdot 10^2$ ($2.3 \cdot 10^1$)	$1.2 \cdot 10^2$ ($1.9 \cdot 10^1$)	$1.2 \cdot 10^2$ ($2.3 \cdot 10^1$)	$1.2 \cdot 10^2$ ($2.3 \cdot 10^1$)
temperature [°C]	$3.7 \cdot 10^1$ ($6.7 \cdot 10^{-1}$)	$3.7 \cdot 10^1$ (1.1)	$3.7 \cdot 10^1$ (1.1)	$3.7 \cdot 10^1$ ($7.5 \cdot 10^{-1}$)
SpO2 [%]	$9.7 \cdot 10^1$ (2.2)	$9.7 \cdot 10^1$ (4.0)	$9.6 \cdot 10^1$ (5.1)	$9.7 \cdot 10^1$ (2.3)
		**BGA measurements**		
pCO2 [mmHg]	$3.9 \cdot 10^1$ (5.8)	$4.4 \cdot 10^1$ (9.8)	$4.3 \cdot 10^1$ (9.8)	$4.0 \cdot 10^1$ (7.0)
pO2 [mmHg]	$9.8 \cdot 10^1$ ($3.4 \cdot 10^1$)	$1.0 \cdot 10^2$ ($2.5 \cdot 10^1$)	$1.0 \cdot 10^2$ ($2.5 \cdot 10^1$)	$9.9 \cdot 10^1$ ($3.2 \cdot 10^1$)
sO2 [%]	$9.7 \cdot 10^1$ (1.6)	$9.6 \cdot 10^1$ (2.8)	$9.6 \cdot 10^1$ (3.5)	$9.7 \cdot 10^1$ (1.6)
Hb (BGA) [$g\,dL^{-1}$]	9.7 (1.7)	9.4 (1.7)	9.5 (1.6)	9.5 (1.7)
lactate [$mg\,dL^{-1}$]	$1.6 \cdot 10^1$ ($1.4 \cdot 10^1$)	$2.7 \cdot 10^1$ ($3.4 \cdot 10^1$)	$4.6 \cdot 10^1$ ($5.3 \cdot 10^1$)	$1.5 \cdot 10^1$ ($1.1 \cdot 10^1$)
pH	7.4 ($5.8 \cdot 10^{-2}$)	7.4 ($8.8 \cdot 10^{-2}$)	7.4 ($1.0 \cdot 10^{-1}$)	7.4 ($6.5 \cdot 10^{-2}$)
type BGA	274 arterial<br>7 venous	104 arterial<br>1 venous	56 arterial	358 arterial<br>10 venous
		**organ replacement therapies**		
renal replacement therapy [%]	4	20	28	7
ECMO [%]	1	2	3	1
impella [%]	0	1	4	0
liver replacement therapy [%]	1	2	4	0
		**ventilation parameters**		
invasive ventilation [%]	48	95	93	59
ventilation [%]	23	80	78	34
APRV [%]	0	2	3	0
FiO2 [%]	$3.2 \cdot 10^1$ ($1.0 \cdot 10^1$)	$4.4 \cdot 10^1$ ($1.8 \cdot 10^1$)	$4.4 \cdot 10^1$ ($1.7 \cdot 10^1$)	$3.4 \cdot 10^1$ ($1.3 \cdot 10^1$)
PEEP [mbar]	7.0 (2.3)	8.9 (3.2)	8.3 (3.3)	8.1 (2.9)
P-peak [mbar]	$2.0 \cdot 10^1$ (5.5)	$2.1 \cdot 10^1$ (6.1)	$2.2 \cdot 10^1$ (5.7)	$2.0 \cdot 10^1$ (5.8)
respiratory frequency [min^{-1}]	$1.7 \cdot 10^1$ (4.4)	$1.8 \cdot 10^1$ (5.3)	$1.7 \cdot 10^1$ (5.6)	$1.7 \cdot 10^1$ (5.0)
		**dose of administered vasopressors and inotropes**		
noradrenaline dose [$\mu g\,kg^{-1}\,min^{-1}$]	$4.4 \cdot 10^{-2}$ ($9.4 \cdot 10^{-2}$)	$2.6 \cdot 10^{-1}$ ($2.6 \cdot 10^{-1}$)	$2.7 \cdot 10^{-1}$ ($3.0 \cdot 10^{-1}$)	$7.7 \cdot 10^{-2}$ ($1.4 \cdot 10^{-1}$)
adrenaline dose [$\mu g\,kg^{-1}\,min^{-1}$]	$9.2 \cdot 10^{-4}$ ($1.1 \cdot 10^{-2}$)	$3.7 \cdot 10^{-3}$ ($2.3 \cdot 10^{-2}$)	$8.6 \cdot 10^{-3}$ ($3.2 \cdot 10^{-2}$)	$7.0 \cdot 10^{-4}$ ($1.0 \cdot 10^{-2}$)
vasopressin dose [$Unit\,kg^{-1}\,min^{-1}$]	$3.8 \cdot 10^{-6}$ ($3.2 \cdot 10^{-5}$)	$5.4 \cdot 10^{-5}$ ($1.4 \cdot 10^{-4}$)	$5.2 \cdot 10^{-5}$ ($1.1 \cdot 10^{-4}$)	$1.2 \cdot 10^{-5}$ ($7.3 \cdot 10^{-5}$)
dobutamine dose [$\mu g\,kg^{-1}\,min^{-1}$]	$2.0 \cdot 10^{-1}$ ($9.3 \cdot 10^{-1}$)	$6.1 \cdot 10^{-1}$ (1.9)	1.1 (2.4)	$2.7 \cdot 10^{-1}$ (1.2)

Table S3: Continuation of table S2, including descriptive statistics for laboratory parameters available within the first ten hours of admission to the intensive care unit. Means are presented with standard deviation (SD) in brackets. Abbreviations denote the glomerular filtration rate (GFR), lactate dehydrogenase (LDH), C-reactive protein (CRP), haemoglobin concentration (Hb), and procalcitonin (PCT)

attribute	no sepsis	sepsis	non survivor	survivor
creatinine [$mg\,dL^{-1}$]	1.3 (1.1)	1.9 (1.5)	1.7 ($9.7 \cdot 10^{-1}$)	1.5 (1.3)
GFR [$mL\,min^{-1}$]	$7.3 \cdot 10^1$ ($3.6 \cdot 10^1$)	$4.9 \cdot 10^1$ ($3.4 \cdot 10^1$)	$4.6 \cdot 10^1$ ($2.9 \cdot 10^1$)	$6.7 \cdot 10^1$ ($3.7 \cdot 10^1$)
LDH [$Unit\,L^{-1}$]	$5.4 \cdot 10^2$ ($7.8 \cdot 10^2$)	$6.8 \cdot 10^2$ ($1.6 \cdot 10^3$)	$1.3 \cdot 10^3$ ($2.3 \cdot 10^3$)	$4.8 \cdot 10^2$ ($6.7 \cdot 10^2$)
bilirubin [$mg\,dL^{-1}$]	1.9 (2.4)	2.4 (3.5)	2.7 (3.7)	1.9 (2.4)
CRP [$mg\,L^{-1}$]	$6.6 \cdot 10^1$ ($7.4 \cdot 10^1$)	$2.0 \cdot 10^2$ ($1.1 \cdot 10^2$)	$1.2 \cdot 10^2$ ($9.5 \cdot 10^1$)	$1.1 \cdot 10^2$ ($1.1 \cdot 10^2$)
leukocytes [nL^{-1}]	$1.1 \cdot 10^1$ (5.0)	$1.6 \cdot 10^1$ ($1.1 \cdot 10^1$)	$1.5 \cdot 10^1$ (9.7)	$1.3 \cdot 10^1$ (7.3)
Hb (lab) [$g\,dL^{-1}$]	9.9 (1.9)	9.8 (1.8)	9.6 (1.6)	9.8 (1.9)
platelets [nL^{-1}]	$1.6 \cdot 10^2$ ($8.3 \cdot 10^1$)	$2.1 \cdot 10^2$ ($1.4 \cdot 10^2$)	$1.8 \cdot 10^2$ ($1.2 \cdot 10^2$)	$1.8 \cdot 10^2$ ($1.1 \cdot 10^2$)
hematocrit [%]	$2.9 \cdot 10^{-1}$ ($5.3 \cdot 10^{-2}$)	$3.0 \cdot 10^{-1}$ ($5.5 \cdot 10^{-2}$)	$2.9 \cdot 10^{-1}$ ($5.1 \cdot 10^{-2}$)	$2.9 \cdot 10^{-1}$ ($5.4 \cdot 10^{-2}$)
sodium [$mmol\,L^{-1}$]	$1.4 \cdot 10^2$ (4.3)	$1.4 \cdot 10^2$ (6.0)	$1.4 \cdot 10^2$ (5.9)	$1.4 \cdot 10^2$ (4.9)
potassium [$mmol\,L^{-1}$]	4.5 ($5.3 \cdot 10^{-1}$)	4.7 ($6.2 \cdot 10^{-1}$)	4.7 ($6.8 \cdot 10^{-1}$)	4.5 ($5.4 \cdot 10^{-1}$)
PCT [$ng\,mL^{-1}$]	1.9 (7.7)	$5.2 \cdot 10^1$ ($1.6 \cdot 10^2$)	$2.3 \cdot 10^1$ ($6.3 \cdot 10^1$)	$1.6 \cdot 10^1$ ($9.6 \cdot 10^1$)
